# Prevalence and Risk Factors of *Leptospira* spp. Infection in Backyard Pigs in the State of Paraná, Brazil

**DOI:** 10.3390/tropicalmed8100468

**Published:** 2023-10-06

**Authors:** Giovanna Fernandes dos Santos, Fernando Antônio Moreira Petri, Gabriele Polia Pires, Ana Karolina Panneitz, Eduarda Ribeiro Braga, Clarisse Sena Malcher, Anna Claudia Baumel Mongruel, João Humberto Teotônio de Castro, Luís Antônio Mathias, Luís Guilherme de Oliveira

**Affiliations:** 1 School of Agricultural and Veterinarian Sciences, São Paulo State University (Unesp), Jaboticabal 14884-900, Brazil; giovanna.f.santos@unesp.br (G.F.d.S.); fernando.petri@unesp.br (F.A.M.P.); g.pires@unesp.br (G.P.P.); ana.panneitz@unesp.br (A.K.P.); eduarda.braga@unesp.br (E.R.B.); cs.malcher@unesp.br (C.S.M.); anna.mongruel@unesp.br (A.C.B.M.); la.mathias@unesp.br (L.A.M.); 2 Paraná Agribusiness Defense Agency (ADAPAR), Curitiba 80035-050, Brazil; joaoteotonio@adapar.pr.gov.br

**Keywords:** leptospirosis, MAT, rural areas, zoonosis

## Abstract

**Simple Summary:**

Leptospirosis is a zoonotic disease that has been increasingly reported around the world. A variety of domestic and wild animal species can serve as natural or accidental hosts for the pathogenic *Leptospira* spp. Among these, swine can function as either maintenance or accidental hosts. This study aimed to investigate the prevalence of *Leptospira* spp. infection and associated risk factors in backyard pigs in the state of Paraná, Brazil. Serological analysis was performed on 1393 serum samples collected from pigs on 188 subsistence properties in different regions of the state. The samples were tested using the microscopic agglutination test (MAT) to detect antibodies against 24 different *Leptospira* spp serovar. The results showed an overall seroprevalence of 68.78% for *Leptospira* spp. antibodies, with the most commonly detected serogroups being Icterohaemorrhagie (19.58%), Pyrogenes (7.94%), and Pomona (7.94%). The lack of rodent control has been identified as a risk factor for *Leptospira* spp. infection in the backyard pig population. This study highlights the high prevalence of *Leptospira* spp. infection in backyard pigs’ sites in Paraná and emphasizes the importance of implementing measures to control and prevent the spread of this zoonotic disease.

**Abstract:**

Leptospirosis is a zoonotic disease that poses a significant threat to human and animal health worldwide. Among different animal species, pigs are known to play a crucial role in the transmission of the pathogenic *Leptospira* spp. This study aimed to investigate the prevalence of *Leptospira* spp. infection and associated risk factors in backyard pigs in the state of Paraná, Brazil. A set of 1393 blood samples were collected from pigs on 188 subsistence properties from 136 different municipalities of the Paraná state and tested using the microscopic agglutination test (MAT) to detect antibodies against 24 different *Leptospira* spp. serovars. The results revealed an overall seroprevalence of 15.87% (221/1393; 95% CI: 13.95–17.78%) for *Leptospira* spp. antibodies, with Icterohaemorrhagiae, Butembo, and Pomona being the most commonly detected in serovar levels. The lack of rodent control (OR 1.12, 95% CI: 0.63–1.98, *p* = 0.02) was the only variable associated with disease incidence and was identified as a significant risk factor for *Leptospira* spp. infection in this context. These findings highlight the urgent need to implement effective control measures, such as improved housing conditions, rodent control, and veterinary assistance, to prevent the spread of this zoonotic disease in backyard pigs in Paraná, Brazil.

## 1. Introduction

Leptospirosis is a bacterial disease caused by multiple species of the *Leptospira* genus [1]. The diseases affects various animal species [2], including pigs, and can be transmitted to humans through contact with water or soil contaminated with the urine of infected animals [3]. The disease is caused by different serovars of the spirochete bacteria that are morphologically and physiologically similar; however, they react to different antigens, all of which belong to the genus *Leptospira* spp., which is distributed almost worldwide [4,5]. Reservoir animals harbor leptospiras in their kidneys for a long time, often without clinical signs, shedding them in the environment through urinary elimination and playing an important role as a source of infection [6,7]. Animals can present variable clinical signs, such as fever, renal and hepatic failure, and reproductive disorders [8,9]. Concerning the association of serovar–reservoir, many epidemiological studies have reported that specific animal species might act as the reservoir for particular *Leptospira* serovars [10]. For example, swine act as a maintenance host for leptospires and are a possible source of human and domestic animal infections [8].

Leptospirosis infections in pigs can result in various clinical signs, including fever, jaundice, loss of appetite, lethargy, and abortion in pregnant females [11]. Additionally, maintenance hosts generally do not develop clinical forms of the disease but act as natural pathogen sources, highly influencing *Leptospira* spp. epidemiology [11,12,13]. The presence of the disease results in economic losses in pig farming [2], as reproductive failures can occur, such as fetal death, abortion in the early term of gestation, infertility, and the birth of weak piglets [14]. Historically, pigs act as a maintenance host for the serovars Bratislava, Pomona, and Tarassovi, while, among the incidental serovars, the most important in pigs are those belonging to the Australis, Icterohaemorrhagiae, Canicola, and Grippotyphosa serogroups [8,15,16]. 

Diseases with zoonotic potential require attention and control due to the risk they pose to the One Health approach. In Brazil, leptospirosis is a disease that must be notified monthly to the Official Veterinary Service as established by normative instruction nº 50 24 September 2013 of the Ministry of Agriculture, Livestock, and Food Supply (MAPA) [17]. The MAPA has programs and policies to encourage the production of subsistence pigs on small rural properties, including funding and technical training programs. However, animal health promotion and disease prevention, such as for leptospirosis, must be considered priorities to ensure food safety and public health. The microscopic agglutination test (MAT) is a widely used method for the serological diagnosis of leptospirosis, particularly for epidemiological research, as it allows for the simultaneous detection of various serovars that belong to different serogroups [2,11]. The test is based on the principle of antigen–antibody reaction and can identify both IgM and IgG antibody classes [18].

Individuals in different occupations that involve direct contact with animals, such as rural workers, animal handlers, veterinarians, and slaughterhouse workers, may contract the disease or become seropositive for some *Leptospira* spp. serovars [19,20,21]. The disease is primarily transmitted to humans through the urine, blood, saliva, and semen of infected animals, particularly rodents [10,22]. Additionally, the lack of effective sanitary measures and promiscuity among animal species have been shown to facilitate the spread of the pathogen in pig farming [23,24]. In subsistence pig farming, where animals are raised on a small scale for self-consumption or local sale, leptospirosis can be a significant concern due to inadequate hygiene and management conditions [25].

Leptospirosis occurs in Brazil with varying frequencies and varies according to the region studied, as reported by Delbem et al. [24]. Several serovars of *Leptospira interrogans* have been associated with infections in pigs from commercial farms. In a study conducted by Favero et al. in 2002 [26], seroprevalences of 33.4%, 50%, and 66.6% were reported for the serovars Grippotyphosa, Autumnalis, and Pomona, respectively, across 10 different Brazilian states, including Bahia, Maranhão, Minas Gerais, Rio de Janeiro, Paraná, Goiás, Santa Catarina, São Paulo, Ceará, Rio Grande do Sul, and Pernambuco, while Ramos et al. [27] reported infections with the serovars Pomona, Copenhageni, Tarassovi, Hardjo, Bratislava, and Wolffi in 18 technified pig farms with reproductive disorders in Rio de Janeiro. Finally, Azevedo et al. [28] reported a seroprevalence of 29% for the serovar Pomona in serum samples from swine slaughtered in Paraiba State in 2008. Both studies reported a high prevalence of the serovar Pomona, which was gradually replaced with Icterohaemorragiae in parallel with advances in improving procedures aimed at controlling rodents and adopting strategies to improve biosecurity as part of the management of Brazilian industrial pig farms, as reported by Petri et al. [29].

To the best of our knowledge, there are limited data on the epidemiology of swine leptospirosis in rural backyard pig sites in the literature. A study conducted in the state of Paraná evaluated the prevalence and risk factors associated with *Leptospira* spp. infection in 344 pigs raised on 86 rural properties [30], where the Copenhageni and Hardjo serovars were the most prevalent. In summary, Leptospira infection in pigs raised in non-technified sites poses a significant problem in Brazil, especially in rural and family agriculture areas. Preventing the disease is crucial to ensure animal health and food safety. Hence, the objective of this study was to examine the seroprevalence of swine leptospirosis in the state of Paraná, Brazil, while considering the associated risk factors and geospatial distribution, given the occurrence of this type of animal rearing.

## 2. Materials and Methods

### 2.1. Sample Design and Study Area

**Figure 1:**
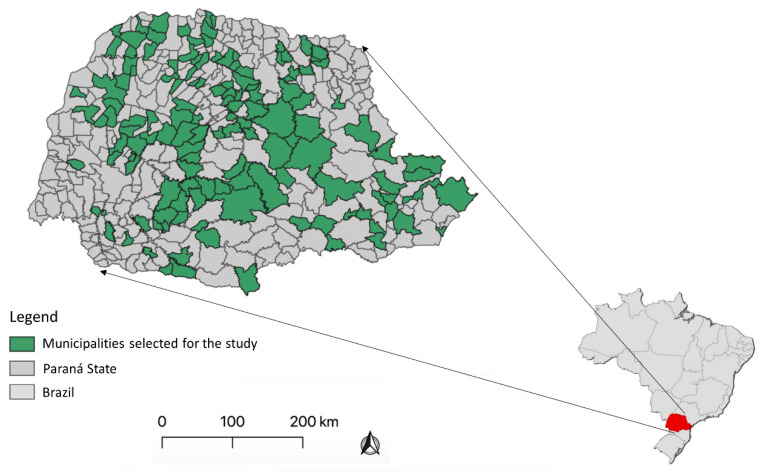
Locations of the sampled backyard pigs by geographical coordinates.

To conduct this study, the Agricultural Defense Agency of Paraná (ADAPAR) provided support in selecting 188 subsistence (Table 1) pig farms (SPF) from 136 different municipalities in the state of Paraná, Brazil. These farms were characterized as non-technical subsistence farms, and samples were collected by the ADAPAR team between January and March 2020. To ensure the reliability of the results, only farms with at least five adult pigs were included in the study, and a minimum distance of five kilometers was determined between the selected farms to minimize the risk of cross-contamination. Figure 1 provides a visual representation of the selected municipalities.

The number of animals used in the study followed non-probabilistic convenience sampling, using as a selection criterion only adult pigs over 8 months of age or already of reproductive age, with sampling per property not less than 46% of the total number of adult pig animals. The total number of samples was determined using the EpiInfo software, considering a total population size of 4917 animals. The estimated expected frequency was 78.6%, based on the findings from the research of Leite (2018) [30]. To achieve a 95% level of reliability within a margin of expected error of 5%, with a design effect of 1 and considering clusters of 188, it was necessary to collect a minimum of two samples per property, totaling 376 serum samples. The farms that were sampled are listed in Table 1.

The blood samples were collected from the jugular vein using sterile disposable syringes and needles and deposited in vacuum tubes free of anticoagulant and with a clot activator (BD^®^ Franklin Lakes, NJ, USA). The blood serum was separated via centrifugation at 1500× *g* for 10 min and aliquoted into plastic graduated microtubes (Eppendorf^®^, Hamburg, Germany). The samples were transported to the Swine Medicine Laboratory at FCAV/Unesp Jaboticabal, where they were transferred to a −20 °C freezer until further processing.

Sample collection was carried out for laboratory analyses and did not involve any suffering of the sampled animals. All methods were carried out in accordance with relevant guidelines and regulations. The research procedures were submitted for approval to the Ethics Committee on the Use of Animals (CEUA) of FCAV/Unesp Jaboticabal under protocol #21/001469.

### 2.2. Epidemiological Questionnaire Application

To obtain epidemiological data on the properties, the owners completed a questionnaire that pertained to potential pigs and property-level risk factors. The questionnaire was based on Loeffen et al. [31] and comprised mostly dichotomous questions with possible answers limited to “yes” or “no”. The questionnaire covered demographic information such as the sex, age, and role (sow or boar) of the pigs, and the risk factors analyzed were SPF located in the settlement, proximity to peri-urban areas, extensive breeding, washing as a component of animal feed, proximity to landfills, proximity to nature reserves, and contact with pigs.

### 2.3. Microscopic Agglutination Test (MAT)

For the serological detection of *Leptospira* spp., the OIE guidelines were followed to perform the MAT, a standardized technique for the serological diagnosis of the pathogen [32,33]. This technique is performed using a collection of live antigens from *Leptospira* spp. that includes 22 pathogenic serovars (Australis, Bratislava, Autumnalis, Butembo, Castellonis, Bataviae, Canicola, Whitcombi, Cynopteri, Grippotyphosa, Hebdomadis, Copenhageni, Icterohaemorrhagiae, Javanica, Panama, Pomona, Pyrogenes, Hardjo, Wolffi, Shermani, Tarassovi, and Sentot) and 2 non-pathogenic serovars (Andamana and Patoc), as adopted by Petri et al. [29]. For laboratory analyses, the 24 serovars identified above, which were, in turn, cultured in Leptospira Medium Base EMJH (Difco™, Franklin Lakes, NJ, USA) for approximately 4 to 8 days and at a concentration of approximately 2.0 × 10^8^ bacteria/mL, determined via counting in a dark-field microscope, were used. The principle behind MAT is simple, but it requires maintaining a panel of live leptospires, representing a biological risk and restricting its practice to specialized laboratories [34].

Briefly, a 1:50 mixture of Phosphate Buffered Saline (PBS 1×, pH 7.4; Sigma-Aldrich, Darmstadt, Germany) solution and blood serum was prepared. In addition, an aliquot of the serovar of *Leptospira* spp. from the culture in the EMJH medium was added to react with the serum to be tested. At the end, 24 serovars were tested in each blood serum sample. Homogenization was performed, the plate was placed in an incubator at 37 °C, and the reaction was read after 40 min. The final dilution of the serum–antigen mixture is defined as 1:100 and is considered the reaction threshold (cut-off). The sera that showed 50% of agglutinated leptospiras under the dark-field microscope were titrated with their respective antigens in a geometric series of dilutions of ratio 2. In the screening, the final titer was given as the reciprocal of the highest dilution at which agglutination occurred [35], in which each sample was classified as reactive or non-reactive with the aid of a dark-field microscope with phase contrast. Grossly, a serum sample was considered positive when the serovar displayed a 50% agglutination titer at the highest dilution, indicating it as the predominant serotype. Conversely, a serum was considered non-reactive when there was no agglutination observed with any of the listed serovars.

### 2.4. Data Analysis

The seroprevalence values obtained for each serovar tested were considered with a 95% confidence interval calculated using the Wilson method. We used Pearson’s chi-square test (*p* < 0.05) to investigate possible associations between the variables in the property characterization questionnaire and the occurrence of *Leptospira* spp. seropositive animals, with each herd considered a sampling unit. If significant differences in exposure frequency were identified between individuals with the disease and those without, we calculated the herd-level relative risk estimate (odds ratio) along with a 95% confidence interval. Variables with *p* < 0.2 in their univariate analysis were submitted to multivariate logistic regression. The analyses were performed using the EpiInfo software (version 7.2.2.6-CDC, Atlanta, USA). Lastly, the calculation of the percentage of reactive samples was performed by dividing the total number of positive reactors by the total number of samples and multiplying this value by 100.

$$Prevalence=\frac{Positive\;samples}{Total\;of\;samples}\times 100$$

## 3. Results

### 3.1. Occurrence of Antibodies against Anti-Leptospira spp.

In total, among the 1393 serum samples tested, 221 were identified as reagents with a titer of at least 1:100 for one of the 24 serovars of *Leptospira* spp. tested. The prevalence of anti-*Leptospira* spp. in the properties sampled was found to be 15.87% (221/1393; 95% CI: 13.95–17.78%). Among the 188 properties that underwent testing, 92 contained at least one reagent sample, resulting in a prevalence of 48.94% (95% CI: 38.70%–59.17%). Table 2 provides the results of the reactive samples by serogroup, serovar, and antibody titer, along with the corresponding prevalence and 95% confidence interval. The most prevalent serogroups in the study were Icterohaemorrhagiae with a prevalence of 5.24% (95% CI: 3.88–6.17%), Pyrogenes with a prevalence of 1.72% (24/1393; 95% CI: 1.04–2.41%), and Pomona with a prevalence of 1.65% (23/1393; 95% CI: 0.98–2.32%). By serovar-level, the most prevalent were Icterohaemorrhagiae with a seroprevalence of 4.88% (68/1393; 95% CI: 3.75–6.01%), Butembo with a prevalence of 1.72% (24/1393; 95% CI: 1.04–2.41%), and Pomona with a prevalence of 1.65% (23/1393; 95% CI: 0.98–2.32%). None of the animals sampled showed antibodies against the serovars Australis, Autumnalis, Bataviae, Cynopteri, Javanica, Pyrogenes, Shermani, Sentot, or Andamana.

### 3.2. Distribution of Animals Seropositive for Leptospira spp.

Based on the MAT and analysis of the data collected, it was found that the most commonly occurring serogroup of *Leptospira* spp. was Icterohaemorrhaegiae with a prevalence rate of 19.58% (95% CI: 14–25.36%). The serogroups Pyrogenes and Pomona followed with prevalence rates of 7.94%. When considering specific serovars, the most common was Icterohaemorrhagiae, with a prevalence of 17.99%. Furthermore, both Butembo and Pomona were detected in 15 different properties, each presenting a prevalence of 7.94%. These findings are summarized in Table 3.

**Figure 2:**
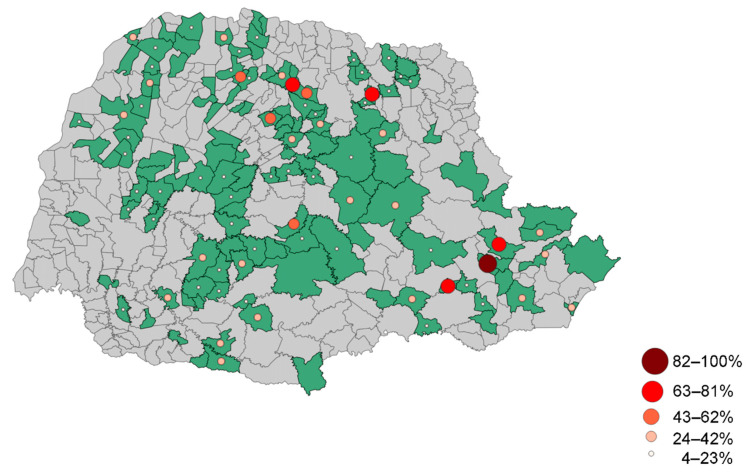
Prevalence of animals that presented seropositivity to serovars of *Leptospira* spp. per municipality.

For a comprehensive perspective on the distribution of the seroprevalence in animals against *Leptospira* spp., Figure 2 presents this information based on municipalities. Additionally, Table 3 provides a breakdown of the serovar prevalence in relation to the number of properties housing seroreactive animals. In total, the study found an overall *Leptospira* spp. prevalence rate of 68.78%.

### 3.3. Risk Factors Associated with Leptospira spp. Antibodies

After collecting and analyzing the data from the epidemiological questionnaires completed by farmers in the study area, none of the variables were found to be potential risk factors for the presence of animals testing positive for anti-*Leptospira* spp. antibodies. This lack of a significant association with disease occurrence persisted even in the bivariate analysis involving all independent variables conducted using the chi-square test (Table 4). However, an exception to this was observed for the variable “lack of rodent control”, as highlighted in Table 5, which provides the risk ratio, 95% confidence interval, and *p*-value for each analyzed risk factor.

## 4. Discussion

The detection of serogroups using MAT is dependent on the phase of the infection being investigated [1]. During the first phase of infection, low antibody titers against common antigens of *Leptospira* spp. and the cross-reactivity of serogroups are typical [1]. Titers of 1:100 or 1:200 may suggest an early stage of infection or residual antibodies from previous infections, while high titers are more common in recent or acute infections [36]. In our study, the low titers observed in most samples could suggest recent exposure to *Leptospira* spp., as shown in Table 2. Furthermore, the presence of positive sera reactions to two serovars at the same time may indicate cross-reactivity and confirm the first phase of infection, but cross-reactivity can also occur between related serovars/serogroups at later stages, as antibodies against common antigens of *Leptospira* are frequently induced during the acute phase of infection [37]. However, a serum positive for a single pathogenic serovar and Patoc cannot be considered as such. Furthermore, it is important to note that carriers, even in low numbers, can rapidly disseminate the pathogen throughout the herd, especially in a suitable (humid) environment for pathogen development [13,30,38]. The severity of *Leptospira* spp. infection detection is generally directly proportional to the number of serovars used in the testing panel. On the other hand, increasing the number of serovars may decrease the specificity of the diagnosis. It is crucial to bear in mind that the majority of infected animals do not present clinical signs, which restricts the application of those concepts solely to the detection of exposure to the etiological agent, rather than confirming the presence of the disease [16].

Furthermore, variations in methodological procedures among laboratories hinder comparisons and the establishment application of sensitivity and specificity values. Consequently, obtaining universally applicable sensitivity and specificity values becomes challenging [16,39].

Determining whether a herd is considered positive based on the presence of a seropositive animal requires the consideration of various parameters, including the sensitivity and specificity of the test used and relevant information at the herd level. This information encompasses the epidemiological context, disease prevalence in the population, disease transmissibility, the presence of other clinical signs or laboratory findings reported by the producers, and other factors relevant to defining the herd’s positivity status.

Based on our findings, it is evident that leptospirosis is a significant public health concern in the state of Paraná, particularly for those involved in subsistence agriculture. The high prevalence of seroreactivity (68.78%) suggests that *Leptospira* spp. can be present in subsistence farms, even those that do not exclusively rear pigs. We observed variation in the identified serogroups (including Icterohaemorrhagiae, Pyrogenes, Pomona, Tarassovi, Castellonis, and others), which indicates that there are multiple sources of infection. However, Icterohaemorrhagiae (19.58%) and Pyrogenes and Pomona (7.94%) were the most commonly detected serogroups (Table 3). The results concerning the seroprevalence of anti-*Leptospira* spp. antibodies are essential to understand the epidemiology of infections caused by the pathogen in non-technical properties because animals are not vaccinated; thus, the detection of antibodies is associated with infection.

A study by Leite et al. (2018) [30] in the state of Paraná assessed the prevalence and risk factors associated with *Leptospira* spp. infection in pigs raised in non-technified systems. The study involved a serological MAT performed on serum samples from 344 pigs across 86 rural properties, and the results revealed a 26.5% prevalence of seropositive pigs for *Leptospira* spp. In the study, the most notable serogroups observed were Icterohaemorrhagiae and Sejroe, which included the pathogenic serovars Copenhageni and Hardjo, respectively. The risk factors associated with infection included the presence of other animal species on the property, pigs’ access to water from streams and creeks, and inadequate hygiene and sanitary management. A similar study was also conducted in the state of Pernambuco [40] that found that the most common serovars associated with infections in pigs were Icterohaemorrhagiae (39.1%), Pomona (25.9%), and Shermani (14.0%). The risk factors associated with infection included stagnant water sources, farms where healthy animals were bred with sick ones, and properties with flooded areas.

The prevalence of the serovar Icterohaemorrhagiae varies across different animal species. While the bacteria may be more commonly found in pigs than in cattle or horses in some cases, the situation may be reversed in other regions or rearing conditions [11,41,42]. Our findings show the significance of this serovar, being the most found serovar with a prevalence of 17.99% and a presence in 26.15% of properties. In certain regions, the prevalence of the serovar Pomona, among other species, may exceed that of the serovar Icterohaemorrhagiae, leading to a greater number of leptospirosis cases in pigs, cattle, and horses [43,44]. Gaining insight into these differences in prevalence is essential to comprehend the epidemiology of leptospirosis and devise appropriate prevention and control strategies.

In subsistence pigs, Pomona is an important serovar, as it can cause reproductive problems, such as abortion and stillbirths, as well as other clinical signs, such as fever and anemia. Pigs are recognized as natural carriers of the bacterium, excreting it in their urine and leading to environmental contamination [3]. This contamination can also impact various other species, including dogs, cattle, sheep, and cats [45,46,47,48,49,50]. Incidental infections of ruminants can occur due to co-grazing with other species, leading to outbreaks and severe clinical illness, while infections via adapted strains are usually chronic and only exhibit mild clinical signs, particularly with regards to reproductive function [50]. In humans, infection with Pomona and other serovars can occur through contact with contaminated animal tissues or fluids, leading to a range of symptoms from mild, flu-like illness to severe disease [51].

The risk factors strongly suggest that rodent control is critical in determining the presence or absence of animals with anti-*Leptospira* spp. Antibodies. The variable “lack of rodent control”, analyzed via univariate analysis, indicated that animals in areas without proper health control were more likely to be exposed to the etiological agent. While the absence of rodent control may contribute to the observed presence of animals with anti-*Leptospira* spp. Antibodies, other variables, such as “Rural settlements or indigenous reservations”, “Peri-urban areas or underserved communities”, “Extensive pig farming”, “Human food waste for animal feed”, “Closeness to landfill sites”, “Closeness to nature reserves”, and “Contact with wild pigs”, may not have a significant impact in this specific context. Nevertheless, our findings revealed that 29.79% (56/188) of properties in the study provided human food waste for pig feed, leading to significant health issues for the animals, such as type C botulism [52]. It is essential to note that the lack of significant differences among the variables tested in the univariate analysis does not necessarily indicate that these conditions are absent or do not play a role in the disease epidemiology.

To effectively combat the spread of *Leptospira* spp. Infection in backyard pigs, implementing a series of crucial measures is imperative. These include improving housing conditions, controlling rodent populations, and providing veterinary assistance. Additionally, it is essential to educate pig farmers and other stakeholders on the importance of the early detection, treatment, and prevention of *Leptospira* spp. Infection in pigs to minimize the risk of zoonotic transmission to humans and other animal species. Overall, this study highlights the urgent need for robust surveillance and control strategies to prevent and control the spread of *Leptospira* spp. Infection in backyard pig sites in Paraná, Brazil. Furthermore, this research emphasizes the value of employing One Health approaches to address zoonotic diseases, such as leptospirosis, which can have significant implications for both human and animal health.

## 5. Conclusions

This study has provided valuable insights into the substantial prevalence of *Leptospira* spp. Infection at backyard pig sites in the state of Paraná, Brazil. The high seroprevalence of *Leptospira* spp. Antibodies in pigs indicates a considerable risk of zoonotic transmission to humans and other animal species. Moreover, the identification of significant risk factors, such as the lack of rodent control, underscores the need for effective control measures to prevent and control the spread of leptospirosis in backyard pigs. These control measures are crucial for safeguarding both human and animal health in the region.

## Figures and Tables

**Table 1:** Number of farms selected based on the sampled herd size in the state of Paraná, Brazil.

Number of Adult Pigs in the Selected Farm	Number of Pig Farms
From 5 to 15	178
From 16 to 20	7
From 21 to 30	2
From 31 to 50	1

**Table 2:** Reactive samples were determined using the microscopic agglutination test (MAT) for each serovar of *Leptospira* spp., along with their respective titers, the prevalence of infection, and a 95% confidence interval in backyard pig sites in the state of Paraná, Brazil.

Serogroup	Serovar	Titer				Total of Reagent Samples	Occurrence (%)	95% CI
		100	200	400	800			
Icterohaemorrhagiae	Icterohaemorrhagiae	68				68	4.88	3.75–6.01
	Copenhageni	5				5	0.36	0.04–0.67
Pyrogenes	Butembo	20	3	1		24	1.72	1.04–2.41
Pomona	Pomona	17	2		4	23	1.65	0.98–2.32
Australis	Patoc ^1^	10	4			14	1.01	0.48–1.53
Canicola	Bratislava	10	3			13	0.93	0.43–1.44
	Canicola	12				12	0.86	0.38–1.35
Tarassovi	Tarassovi	13				13	0.93	0.43–1.44
Sejroe	Wolffi	3		6	1	10	0.72	0.27–1.16
	Hardjo	2				2	0.14	0.00–0.34
Whitcombi	Whitcombi	10				10	0.72	0.27–1.16
Castellonis	Castellonis	4	5			9	0.65	0.22–1.07
Hebdomadis	Hebdomadis	6				6	0.43	0.09–0.77
Grippotyphosa	Grippotyphosa	6				6	0.43	0.08–0.77
Panama	Panama	6				6	0.43	0.08–0.77
Total		192	17	7	5	221	15.87	13.95–17.78

^1^ Non-pathogenic serovar.

**Table 3:** Prevalence of *Leptospira* spp. serovars per property and 95% confidence interval at backyard pig sites in the state of Paraná, Brazil.

Serogroup	Serovar	Total of Properties with Reagent Animals	Occurrence (%)	CI 95%
Icterohaemorrhagiae	Icterohaemorrhagiae	34	17.99	12.51–23.47
	Copenhageni	3	1.59	0.00–3.37
Pyrogenes	Butembo	15	7.94	4.08–11.79
Pomona	Pomona	15	7.94	4.08–11.79
Australis	Patoc ^1^	9	4.76	1.73–7.80
Tarassovi	Tarassovi	9	4.76	1.73–7.80
Castellonis	Castellonis	8	4.23	1.36–7.10
Canicola	Bratislava	7	3.70	1.01–6.40
	Canicola	7	3.70	1.01–6.40
Sejroe	Wolffi	6	3.17	0.68–5.67
	Hardjo	1	0.53	0.00–1.56
Whitcombi	Whitcombi	6	3.17	0.68–5.67
Grippotyphosa	Grippotyphosa	5	2.65	0.36–4.93
Panama	Panama	3	1.59	0.00–3.37
Hebdomadis	Hebdomadis	2	1.06	0.00–2.52
Total		130	68.78	62.18–75.39

^1^ Non-pathogenic serovar.

**Table 4:** Bivariate analysis of all independent variables and the respective frequency of each risk factor for the occurrence of *Leptospira* spp. antibodies in backyard pig sites in the state of Paraná, Brazil.

Independent Variables	Frequency	*p*-Value
Lack of rodent control	45.40% (85/188) ^a^	0.971
Extensive pig farming	40.96% (77/188) ^a^	
Human food waste for animal feed	29.79% (56/188) ^a^	
Peri-urban areas or underserved communities	24.47% (46/188) ^a^	
Closeness to nature reserves	20.21% (38/188) ^a^	
Rural settlements or indigenous reservations	11.7% (22/188) ^a^	
Contact with wild pigs	9.04% (17/188) ^a^	
Closeness to landfill sites	2.66% (5/188) ^a^	

^a^ Frequencies followed by the same letter are not significantly different by chi-square test (*p* > 0.05).

**Table 5:** Association between leptospirosis prevalence in municipalities in the state of Paraná, Brazil, with the variables evaluated using logistic regression (*p* = 0.05).

Risk Factor	OR	CI OR (95%)	*p*-Value
Rural settlements or indigenous reservations	0.68	0.27–1.67	0.53
Peri-urban areas or underserved communities	1.45	0.74–2.84	0.35
Extensive pig farming	1.08	0.60–1.94	0.90
Human food waste for animal feed	0.76	0.40–1.42	0.48
Lack of rodent control	1.12	0.63–1.98	0.04
Closeness to landfill sites	0.67	0.11–4.13	1.00
Closeness to nature reserves	0.69	0.34–1.42	0.41
Contact with wild pigs	0.90	0.33–2.45	0.93

## Data Availability

Not applicable.
